# Identification of Immunogenic Cytotoxic T Lymphocyte Epitopes Containing Drug Resistance Mutations in Antiretroviral Treatment-Naïve HIV-Infected Individuals

**DOI:** 10.1371/journal.pone.0147571

**Published:** 2016-01-25

**Authors:** Juan Blanco-Heredia, Aarón Lecanda, Humberto Valenzuela-Ponce, Christian Brander, Santiago Ávila-Ríos, Gustavo Reyes-Terán

**Affiliations:** 1 Centre for Infectious Diseases Research, National Institute of Respiratory Diseases, Mexico City, Mexico; 2 Max Planck Institute for Molecular Biomedicine, Münster, Germany; 3 IrsiCaixa AIDS Research Institute-HIVACAT, Hospital Germans Trias i Pujol, Badalona, Spain; 4 Institució Catalana de Recerca i Estudis Avançats (ICREA)-UAB, Barcelona, Spain; 5 University of Vic and Central Catalonia, Vic, Spain; University of Alabama at Birmingham, UNITED STATES

## Abstract

**Background:**

Therapeutic HIV vaccines may prove helpful to intensify antiretroviral treatment (ART) efficacy and may be an integral part of future cure strategies.

**Methods:**

We examined IFN-gamma ELISpot responses to a panel of 218 HIV clade B consensus-based HIV protease-reverse transcriptase peptides, designed to mimic previously described and predicted cytotoxic T lymphocyte epitopes overlapping drug resistance (DR) positions, that either included the consensus sequence or the DR variant sequence, in 49 ART-naïve HIV-infected individuals. Next generation sequencing was used to assess the presence of minority DR variants in circulating viral populations.

**Results:**

Although a wide spectrum of differential magnitudes of response to DR vs. WT peptide pairs was observed, responses to DR peptides were frequent and strong in the study cohort. No difference between the median magnitudes of response to DR vs. WT peptides was observed. Interestingly, of the 22 peptides that were recognized by >15% of the participants, two-thirds (64%) corresponded to DR peptides. When analysing responses per peptide pair per individual, responses to only WT (median 4 pairs/individual) or DR (median 6 pairs/individual) were more common than responses to both WT and DR (median 2 pairs/individual; p<0.001). While the presence of ELISpot responses to WT peptides was frequently associated with the presence of the corresponding peptide sequence in the patient’s virus (mean 68% of cases), responses to DR peptides were generally not associated with the presence of DR mutations in the viral population, even at low frequencies (mean 1.4% of cases; p = 0.0002).

**Conclusions:**

Our data suggests that DR peptides are frequently immunogenic and raises the potential benefit of broadening the antigens included in a therapeutic vaccine approach to immunogenic epitopes containing common DR sequences. Further studies are needed to assess the quality of responses elicited by DR peptides.

## Introduction

The development of therapeutic human immunodeficiency virus type-1 (HIV-1) vaccines has gained interest in recent years as an aid for treating individuals already infected with HIV-1 as part of HIV-1 cure strategies [1, 2]. Although antiretroviral treatment (ART) can effectively control HIV-1 replication extending the lifespan of HIV-1-infected individuals to levels close to those observed in the general population [3, 4], the therapeutic regimen must be maintained for lifetime, with treatment interruption resulting in immediate viral rebound. In addition, ART alone cannot eliminate HIV-1 infection due to the virus’ ability to persist in individuals through the formation and maintenance of viral latent reservoirs and residual viral replication [5]. This represents a considerable challenge for the patient, implying the need of strict adherence and the possibility of development of long-term drug toxicity and HIV-1 drug resistance. It also challenges public health systems, with the economic and logistic burden of maintaining therapeutic regimens for extended period of times and the need for patient clinical and laboratory monitoring. Considering this scenario, the development of a therapeutic vaccine that could intensify ART efficacy by controlling residual replication or that could even substitute ART all together would be desirable. Moreover, therapeutic vaccination may also provide the means for effective combined HIV-1 cure strategies that are based on purging the latent viral reservoir with latency reversal agents and the removal of reactivated cells by CTL [6].

However, the extraordinary adaptability of HIV-1, associated with high mutation and recombination rates and remarkable replication capability, has complicated the development of effective vaccine candidates. Indeed, it has been well documented that HIV-1 can readily escape from HLA class I-restricted, virus-specific CD8+ cytotoxic T lymphocyte (CTL) responses by the selection of mutations that can prevent proper viral epitope processing, epitope binding to HLA molecules or recognition by specific T cell receptors [7]. Significant advances have been made towards the identification of viral epitopes which elicit strong CTL responses, are common in a significant proportion of the population and are less prone to variation or, if they mutate at all, lead to mutant viruses with significantly reduced viral replicative capacity [8].

We previously proposed the hypothesis that *in vivo* HIV-1 replication and escape from pharmacological and immune control could be limited by the concerted use of opposing selective pressures acting on the virus and that this concept could be used for the selection of putative immunogens for candidate vaccine design [9]. Specifically, we proposed a scenario in which drug resistant variants could be targeted by the host’s virus-specific CTL response. In order to escape from the specific CTL response the virus would have to revert back to wild type, which in turn would be targeted by ART. Thus, in the presence of ART, the virus would be cornered between the immunologic and pharmacologic selective pressures. On the other hand, mutations could also exist that would allow the virus to escape both the CTL response and ART. However, their replication deficiency may reduce the advantage of this double escape for the virus considerably [10]. In the present work, we examined the immunogenicity of putative CTL epitopes within the Pol PR-RT region containing drug resistance mutations, as a proof of concept that the possibility of forcing the virus between opposing selective pressures could exist.

## Materials and Methods

### Ethics statement

The present study was evaluated and approved by the Ethics Committees of the National Institute of Respiratory Diseases in Mexico City and the IrsiCaixa AIDS Research Institute in Barcelona, Spain and was conducted according to the principles of the Declaration of Helsinki. All the participants gave written informed consent before blood sample donation.

### Study subjects

A total of 49 subtype B HIV-1-infected, ART-naïve individuals were enrolled in the present study at the IrsiCaixa AIDS Research Institute in Barcelona, Spain (24/49) and at the Centre for Research in Infectious Diseases (CIENI) of the National institute of Respiratory Diseases in Mexico City (25/49). Blood samples donated by the participants were used to isolate and store plasma and peripheral blood mononuclear cells (PBMCs).

### Peptides

**Fig 1:**
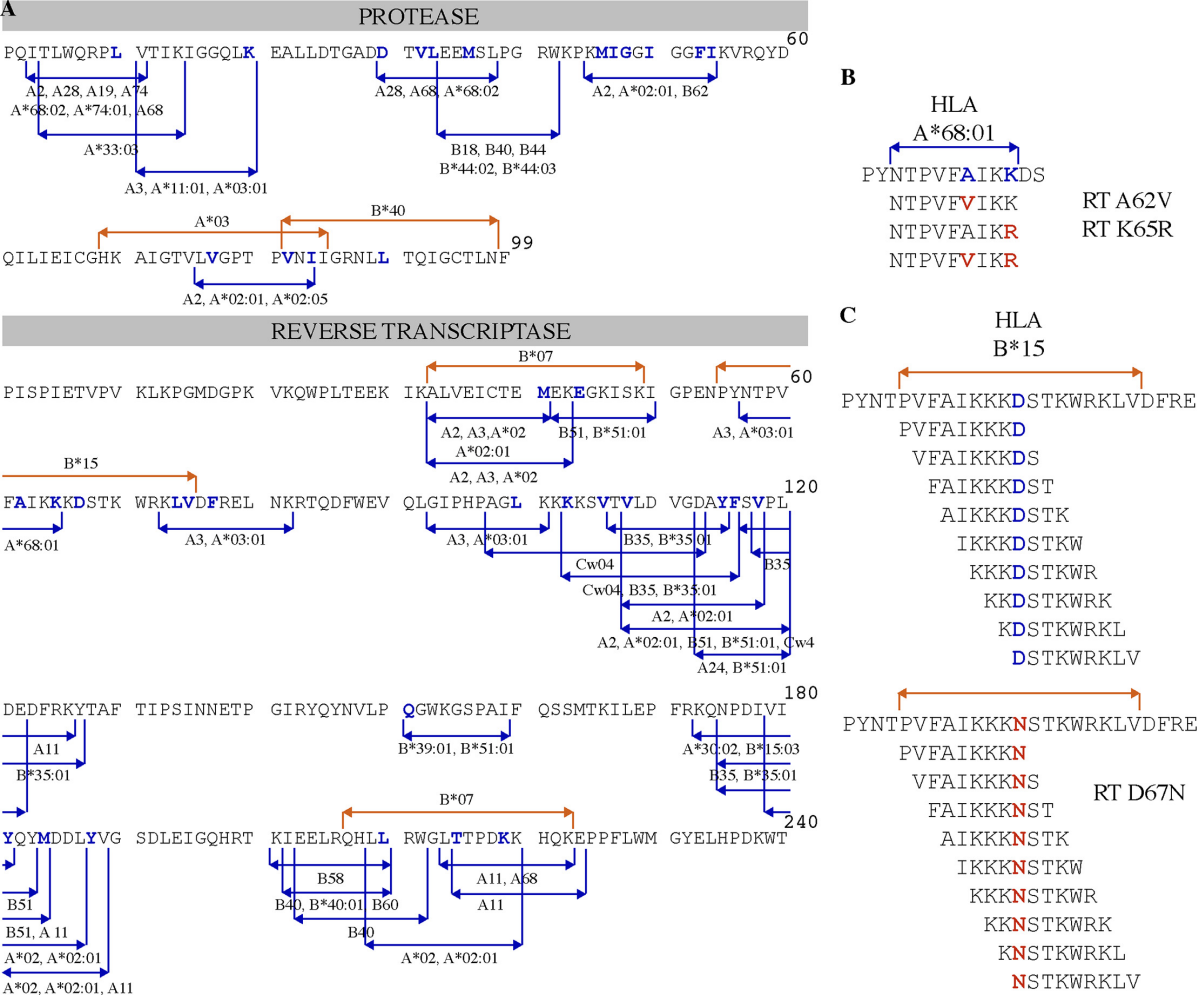
Peptide design for the evaluation of the “cornering hypothesis”. **A.** Pro and RT CTL epitope map used for peptide design. Peptides were designed based on previously-described optimal CTL epitopes (blue lines) and overlapping statistically-predicted HLA-associated positions (red lines) [13]. Specific changes in the WT sequence known to mediate ART resistance are indicated as blue residues. **B.** 128 peptides were designed using experimentally confirmed CTL epitopes that overlap positions associated with DR. Both the WT peptide and the DR variants were synthesized. In the example shown, two DR mutations are reflected in an HLA-A*68:01-restricted epitope and four peptides were design reflecting the different combinations of mutations. **C.** Ninety additional peptides were designed based on 5 regions containing HLA footprints and DR mutation sites reported in [13]. A representative example is shown for the set of peptides designed to test the effect of DR mutation D67N, which is also associated with an HLA-B*15 footprint. Both WT and DR peptides were synthesized and tested individually.

Two sets of peptides adding up a total of 218 were used to screen for HIV-specific T-cell responses. A set of 128 peptides (Set “b”) was designed based on HIV clade B consensus sequence [11] reproducing 33 optimally defined CTL epitopes in HIV protease (PR) and reverse transcriptase (RT), reported at the Los Alamos HIV Immunology Database [12], that include at least one drug resistance site. Individual peptides were synthesized containing either the wild type (WT) amino acid or the drug resistance (DR)-associated amino acid. When multiple DR sites were included in the same epitope, peptides with different combinations of WT and DR amino acids were synthesized (Fig 1A and 1B). A second set of 90 subtype B-based [11] peptides (Set “a”) was designed to study five DR sites on PR-RT which had been previously also associated with HLA selective pressure, but for which no optimal epitopes have been described [13]. For each of these 5 sites, sets of 9-mer peptides overlapping by 8 amino acids were synthesized including the DR site of interest in each one of the positions of the peptide (Fig 1A and 1C). In order to compare immunogenicity, peptides with and without DR mutations were arranged in 140 pairs. For Set “b”, a single WT peptide could have more than one DR peptide counterpart, adding up to 95 peptide pairs; for Set “a”, each of 45 WT peptides was paired with its corresponding DR peptide, adding up to 45 peptide pairs. A complete list of the peptides evaluated can be consulted in S1 Dataset. All peptides were synthesized at JPT Innovative Peptide Solutions (Berlin, Germany).

### ELISpot Assays

PBMCs were isolated from whole blood by density gradient centrifugation (Lymphoprep Axis-Shield Oslo, Norway) within 6 hours of venipuncture and cryo-preserved until use. Frozen cells were thawed, and incubated in RPMI 1640 containing 10% heat-inactivated fetal calf serum (both from Sigma-Aldrich, St. Louis, MO), 2 mM L-glutamine, 50 U of penicillin/ml, 50 μg of streptomycin/ml, and 10 mM HEPES (all from Mediatech, Herndon, VA) (R10). Thawed cells were rested at 37°C and 5% CO_2_ for 4 hours prior to use in ELISpot assays. After resting, cells were counted and plated in 96-well polyvinylidene difluoride plates (Millipore, Bedford, MA), which had been precoated overnight with 0.5 μg/ml anti-gamma interferon (IFN-γ) monoclonal antibody 1-D1K (Mabtech, Stockholm, Sweden). Each peptide was added individually at a final concentration of 14 μg/ml as previously described [14]. A total of 100,000 cells per well were added in 140 μl of R10. For negative controls, cells were incubated in medium alone. Staphylococcal Enterotoxin B Fragment (150–161) (Sigma) was added at a concentration of 7 ng/ml to serve as a positive control in experiments carried out at CIENI and Phytohemagglutinin at a concentration of 10 μg/ml (1:10 dilution Mabtech) in experiments performed at IrsiCaixa. The cells were incubated overnight at 37°C with 5% CO_2_. The plates were then washed six times with phosphate-buffered saline (without Ca or Mg; Mediatech), and 0.5 μg/ml biotinylated anti-IFN-γ monoclonal antibody 7-B6-1 (Mabtech) was added for 1 h at room temperature. The plates were again washed and incubated with a 1:2000 dilution of streptavidin-coupled alkaline phosphatase (Mabtech) for 1 hour at room temperature in the dark. The plates were washed again, and IFN-γ production was detected as dark blue spots after a short incubation with nitroblue tetrazolium and 5-bromo-4-chloro-3-indolyl phosphate (Bio-Rad, Hercules, CA). The color reaction was stopped by washing the plates with tap water. Spots were counted using a C.T.L. Reader unit at IrsiCaixa and a Bioreader 5600 (Bio-Sys, Germany) at CIENI; the results were expressed as spot-forming cells (SFC) per million input cells.

Thresholds for positive responses were determined as at least five spots (50 SFC/10^6^ PBMCs) per well and as responses exceeding the mean of negative wells plus 3 standard deviations or three times the mean of negative control wells, whichever was higher.

### HIV PR-RT PCR Amplification and deep sequencing

Viral RNA was extracted from 1 mL of plasma (QIAamp Viral RNA Kit; QIAGEN, Valencia, CA). A fragment of the viral *pol* gene including the whole protease (PR) and 334 codons of the reverse transcriptase (RT) was amplified by nested RT-PCR with primers PR_5’_OUTER 5’-CCCCTAGGAAAAAGGGCTGTTG-3’ (HXB2 positions 2009–2030) and RT_3’_OUTER 5’- GTTTTCAGATTTTTTAAATGGCTCTTG-3’ (3576–3602) for the first round of amplification, and PR_5’_INNER 5’-TGAAAGATTGTACTGAGAGACAGG-3’ (2057–2080) and RT_3’_INNER 5’-GGCTCTTGATAAATTTGATATGTCC-3’ (3559–3583) for the second round of amplification. SuperScript III OneStep RT PCR (Invitrogen, Brown Deer, WI) and Platinum Taq DNA polymerase (Invitrogen) were used for the first and second round PCR respectively, according to manufacturer’s recommendations with the following conditions: 50°C for 30 min (first round only); 94°C for 3 min; 35 cycles of 94°C for 30 sec, 60°C for 30 sec, 72°C for 2 min and a final extension of 72°C for 5 min.

Semiconductor Next Generation Sequencing (NGS) was performed to test for DR variants within the viral populations of each participant. Briefly, PR-RT PCR products were enzymatically fragmented and used to build barcoded genetic libraries for each virus using the Ion Xpress Plus Fragment Library Kit (Thermo Fisher Scientific, Waltham, MA), according to manufacturer’s instructions. Sequencing template was created by emulsion PCR using the Ion PGM Template OT2 200 Kit (Thermo Fisher Scientific) and sequencing was performed using the Ion PGM Sequencing 200 Kit v2 (Thermo Fisher Scientific), on 316v2 chips using an Ion PGM instrument (Thermo Fisher Scientific). Data from the Ion PGM runs were processed initially using the Ion Torrent platform-specific pipeline software Torrent Suite v1.3.1 to generate sequence reads, trim adapter sequences, filter, and remove poor signal-profile reads. Further analysis consisted of a re-sequencing approach with iterative alignments, adapting the reference for each cycle, and forking reference sequences to account for quasispecies. This yielded a semiquantitative value of the proportion of quasispecies in each sample. Next generation sequencing files are held at the BioSample NCBI database, accession numbers: 4276943 to 4276962.

### HIVDR analysis from NGS runs

The frequency of DR mutations within the viral population for each patient was assessed from NGS runs using HyDRA, an automated HIVDR analysis pipeline developed by the National Microbiology Laboratory of the Public Health Agency of Canada [15]. Briefly, a quality control filter was first applied to each NGS run, selecting reads with length >100 bases and average quality score >Q30. Reads were then mapped against the HXB2 reference sequence using SMALT (Wellcome Trust Sanger Institute, Hinxton, UK), with k-mer size 11 and step size 1. Read mappings with overlap values (percentage of read included in mapping to reference) under 65% and identity values (percentage of read mapping which matched the reference) under 75% were filtered out. Variants were called using a custom viral single nucleotide variant caller, based on a Poisson distribution. Variants were filtered out when quality <Q30, read depth <100 and allele count <5. Amino acid mutations were then called and queried against a merged DR mutation database including the WHO list of mutations for DR surveillance [16] and the Stanford HIVDR Database [17]. All DR mutations with frequency over 1% are reported. Nevertheless, a conservative threshold of 2% was used to define the presence of DR mutations given that the ability to detect minority variants depends on sequencing depth and on viral load. Using our sequencing protocol, a sequencing depth of 1500-2000x and viral load >2500 copies/mL would be needed in order to accurately call variants at 1% frequency.

### Epitope and peptide variant frequency determination using deep sequencing runs

NGS reads were aligned, allowing one mismatch per read using the short read component of the Burrows-Wheeler Aligner (BWA) [18] against the HIV-1 HXB2 reference genome (NCBI accession number K03455.1). Reads with multiple mismatches were discarded. Using a customized R script, all reads containing bases with individual quality scores <Q20 were discarded and the rest were translated according to the standard genetic code and the frame of the gene to which they mapped. Read mappings were then compared against all peptide sequences included in the study. The frequency of read mappings with 100% identity to each peptide sequence assayed was calculated with the same R script and used for further analysis.

### Human Leucocyte Antigen typing

DNA for typing was extracted using the QIAamp DNA Blood (QIAGEN, Valencia, CA) from 6 million PBMC, according to the manufacturer’s instructions. For all samples, HLA class I typing was performed at high resolution by sequence-based typing, using commercial kits (SeCore A, B and Cw Locus Sequencing Kits, Thermo Fisher Scientific, Waltham, MA). Allele assignments were made with uTYPE v6.0 (Thermo Fisher Scientific). Ambiguities were resolved using the HLA Completion tool available on-line [19].

### Statistical Analyses

We performed Mann-Whitney U tests (STATA/SE v12, StataCorp, College Station, TX) to look for associations between the presence/absence of all HLA class I alleles found in the cohort and positive ELISpot responses to each peptide assayed. These associations were corrected for multiple comparisons using false-discovery rate analysis (Storey q-value, q value package for R v2.12). Wilcoxon ranked tests were used to compare the magnitudes of ELISpot responses between WT and DR peptide pairs.

## Results

### Frequent and strong responses are observed to CTL epitopes containing drug resistance mutations

**Fig 2:**
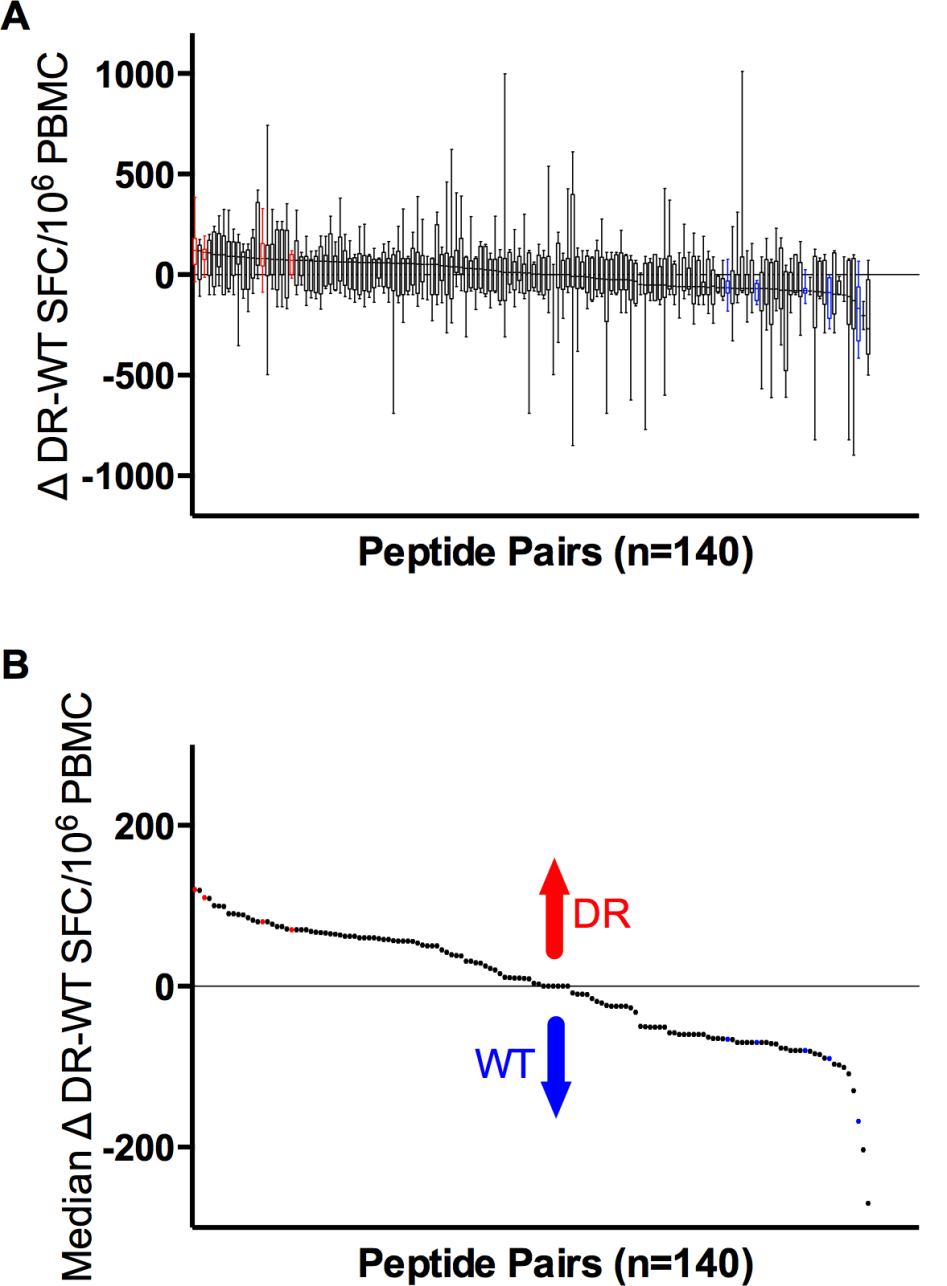
Wide spectrum of differential magnitudes of response to WT and DR peptide pairs. **A.** Differential magnitudes of response to each DR-WT peptide pair (Δ) are shown for each responder. Δ were calculated as the magnitude of response (SFC/million PBMC) to the DR sequence minus the magnitude of response to the WT sequence in each subject individually. A median differential magnitude of response was then calculated across all individuals responding to either the WT, the DR or both sequences. Boxes represent 50% of differential magnitude to each DR-WT peptide pair, whiskers maximum and minimum of the differential magnitude. **B.** Only median Δ responses are shown. Peptide pairs with significantly higher differential response to DR are shown in red; peptide pairs with significantly higher differential response to WT are shown in blue (p<0.05).

Considering that the immune recognition of DR variants might offer an opportunity to impose double selection pressure on HIV, we assessed the T cell response against CTL epitopes overlapping DR positions and regions with HLA class I footprints coinciding with DR positions [13] using peptides containing the WT or DR variant. The 218 peptides tested were arranged in 140 WT-DR pairs (95 corresponding to Set “b” and 45 to Set “a”, as explained in Methods). When multiple DR mutations were present in the same peptide, each DR variant peptide synthesized was compared against the same WT peptide. We determined the frequency of recognition of DR and WT sequences and compared the magnitude of response for each DR–WT peptide pair (n = 140). Of all the 218 peptides tested, 217 (99.5%) were recognized by at least one of a total of 49 subjects tested. The median of patients recognizing each of the 218 peptides was 5 (range 0–15 patients, IQR 4–6). The median number of responses observed per individual was 16 (range 0–123, IQR 1–35). The total number of responses considering all the participants and peptides was 1,120. We observed a wide spectrum of differential magnitudes of response to DR vs. WT peptide pairs (Fig 2). This observation was maintained when dividing the peptide pairs into Set “a” and Set “b”, according to the peptide design rationale (see Methods; S1 Fig). From the 140 pairs analyzed, four (two belonging to Set “a” and two to Set “b”) elicited significantly stronger responses by the DR variant, while five (one belonging to Set “a” and four to Set “b”) showed significantly stronger responses to the WT variant (Table 1; S1 Fig). The DR mutations included in the peptide pairs with stronger responses to the DR variant were L33I, I50V and L90M in the protease and T215Y in RT. These observations suggest that some DR variants are strongly immunogenic in a large number of individuals tested in the present cohort. Moreover, these observations suggest that peptide design rationale was not a significant factor influencing differential magnitudes of response to WT and DR peptides.

**Table 1:** Peptide pairs with significant median differential magnitude of response Δ DR-WT ^a^.

WT peptide	Magnitude of response to WT peptide [Median (range); SFC/10^6^ PBMC]	DR peptide	Magnitude of response to DR peptide [Median (range); SFC/10^6^ PBMC]	Median Δ DR-WT response (SFC/10^6^ PBMC)	HIV Gene	DR mutation	p value
RNL**L**TQIGC	0 (0–140)	RNL**M**TQIGC	130 (51–450)	120	PR	L90M	0.0009
QHLLRWGL**T**	0 (0–62)	QHLLRWGL**Y**	115 (60–200)	110	RT	T215Y	0.0005
DTV**L**EEMNL	29 (0–200)	DTV**I**EEMNL	130 (60–380)	80	PR	L33I	0.0020
KMIGG**I**GGFI	0 (0–163)	KMIGG**V**GGFI	80 (51–163)	70	PR	I50V	0.0039
EK**E**GKISKI	72 (56–190)	EK**D**GKISKI	0 (0–113)	-66	RT	E44D	0.0195
**K**KSVTVLDVGDAYFS	123 (51–930)	**N**KSVTVLDVGDAYFS	0 (0–890)	-70	RT	K103N	0.0078
EELRQHL**L**RW	80 (0–164)	EELRQHL**W**RW	0 (0–60)	-80	RT	L210W	0.0007
KKS**V**TVLDVGDAYFS	123 (51–930)	KKS**M**TVLDVGDAYFS	0 (0–690)	-90	RT	V106M	0.0313
NL**L**TQIGCT	170 (0–484)	NL**M**TQIGCT	0 (0–238)	-168	PR	L90M	0.0005

^a^ Wilcoxon Signed Rank Test. Positions associated with drug resistance are shown in bold.

**Fig 3:**
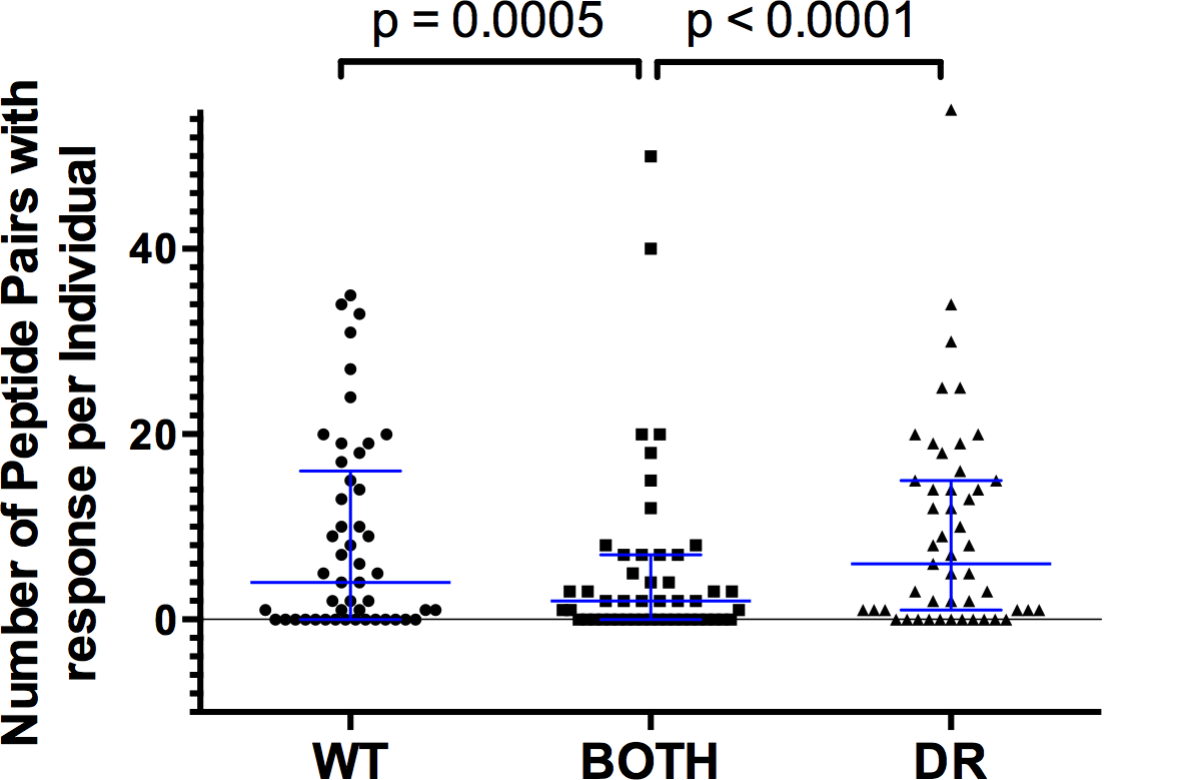
Recognition frequency of DR mutations. ELISpot responses to a panel of 140 DR-WT peptide pairs were assessed in 49 individuals. The number of peptide pairs for which, both WT and DR, only DR, or only WT peptides were recognized was assessed per individual. The scatter plot shows the result of three Wilcoxon tests comparing the paired data between groups, significant p values are indicated. Error bars show the median with interquartile range.

Indeed, as can be seen in Fig 3, a median of 4 peptide pairs per individual showed responses only to WT, a median of 2 peptide pairs per individual showed responses to both WT and DR variants while a median of 6 peptide pairs per individual showed responses only to the DR variant. These observations suggest that the presence of DR mutations can result either in the creation of new CTL epitopes or in the loss of WT epitopes. The data also indicate that DR or WT-specific responses are more common than fully cross-reactive responses (p<0.0001 and p = 0.0005 respectively, Fig 3), in line with the idea that many DR-mutations are effective CTL escape variants [20, 21]. Importantly, these observations were maintained in both peptide sets analysed separately (S2 Fig).

We then investigated if the existence of an HLA association for a specific DR site influenced responses to DR peptides, using peptide Set “b” only. Importantly, few positions in protease and RT, which are associated with DR have also shown association with HLA. The most complete list of associations in the largest cohort analysed so far, composed of ART-naïve HIV B clade-infected mainly Caucasian individuals from North America and Australia [22] included 8 HLA associations that coincide with DR mutations sites (3 in PR and 5 in RT; S1 Dataset). Considering peptide Set “b” (including 95 peptide pairs in total), 39 peptide pairs overlapped HLA-associated DR positions according to Carlson et al., while 56 peptide pairs overlapped DR positions without HLA association. Interestingly, responses to WT or DR peptides were significantly higher than responses to both WT and DR in only peptide pairs overlapping DR positions without HLA association (S3 Fig). Additionally, the distribution of differential magnitudes of responses to peptide pairs overlapping DR positions with and without HLA associations were similar, both groups including peptide pairs with significantly higher responses to WT or DR peptides (S3 Fig).

In order to distinguish real responses to specific peptide sequences from borderline cross-reactivities, the median magnitudes of response to WT and DR peptides were compared (S1 Table). No differences were observed in the median magnitudes of response to WT (median 95, IQR 80–105 SFC/10^6^ PBMC) and DR (median 99, IQR 81–116 SFC/10^6^ PBMC) peptides (p = 0.2844), suggesting that responses observed to DR peptides are not associated with borderline cross-reactivity and represent specific responses. Indeed, in the case of peptide Set “a”, responses to DR peptides were even significantly stronger than responses to WT peptides (p = 0.0125; S1 Table).

### A high proportion of the most frequently recognized peptides in the study cohort include DR mutations

**Fig 4:**
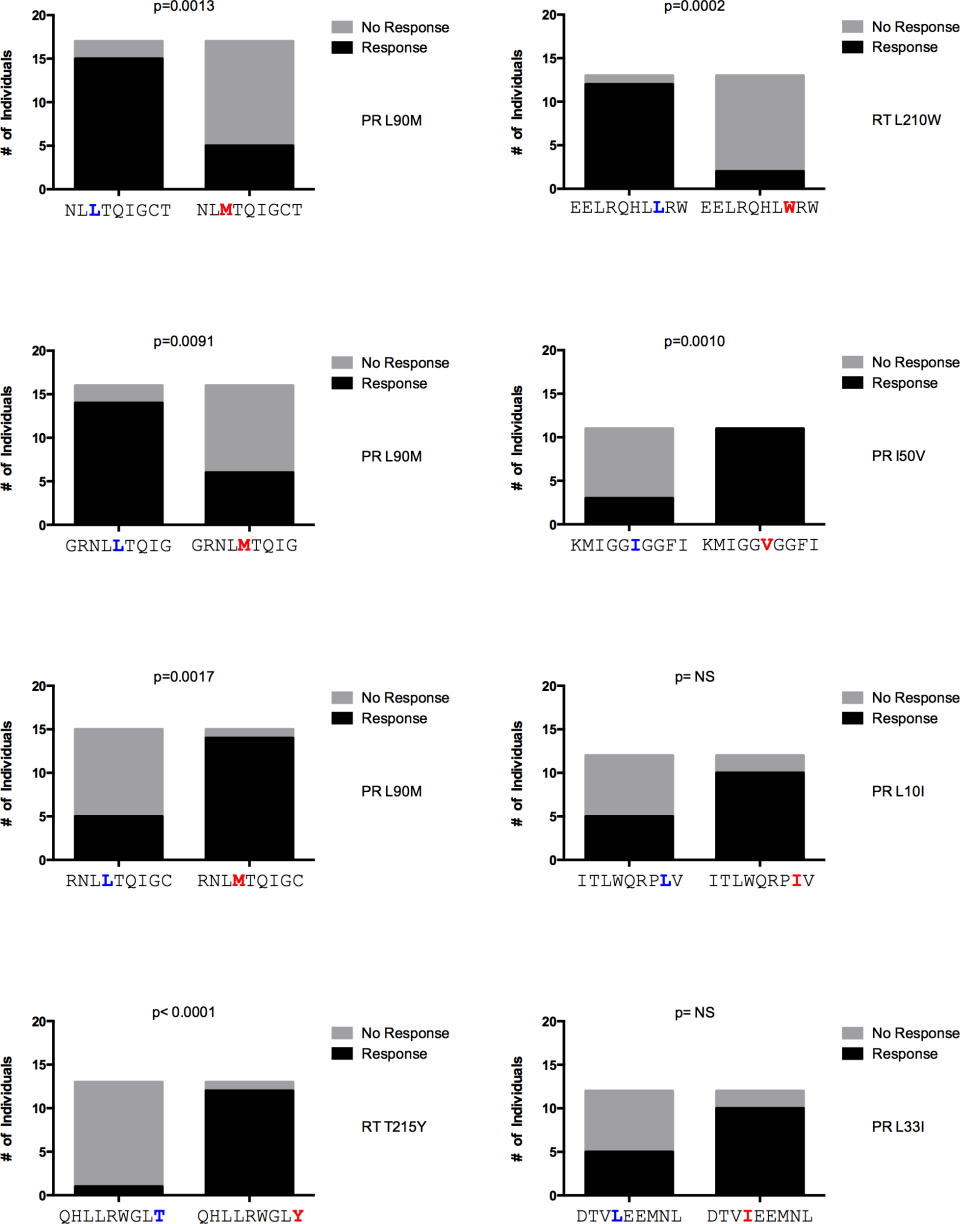
Most frequently recognized peptides in the study cohort. Peptides with IFN-gamma ELISpot responses in 20% or more individuals in the study cohort are shown with their DR or WT peptide pair. These 8 peptides were recognized by at least 10 individuals, giving us sufficient power to compare median magnitude of response between the WT and DR peptide. DR mutations are shown in red. WT amino acids in DR mutations sites are shown in blue. Statistically significant differences (Fisher’s exact test) in recognition of the WT vs. DR peptide were observed for 6 out of the 8 peptide pairs.

Across all responses, we identified 22 peptides (7 from Set “a” and 15 from Set “b”) that were recognized by more than 15% (more than 7 individuals) of the cohort. Fourteen (63%) of these frequently recognized peptides were sequences that reflected DR mutations (4 from Set “a” and 10 from Set “b”; Table 2). Remarkably, eight peptides were recognized by more than 20% (more than 9 individuals) of the cohort, and five of them (63%) included DR mutations (Fig 4). Within these eight cases, recognition of the three WT peptides was statistically higher than their DR counterparts and recognition of three of the five DR peptides was higher than their WT counterparts (Fig 4). Considering the top 5, 10 or 20 most frequently recognized peptides, the proportion of peptides reflecting DR mutations was 40%, 50% and 65% respectively. Taken together, these observations suggest that specific responses to DR peptides are highly frequent in the study cohort.

**Table 2:** Most frequently recognized peptides.

Peptide ^a^	Peptide Set ^b^	WT or DR	DR position overlapped	HIV Protein	Individuals responding to peptide [n (%)]	Median magnitude of responses (range; SFC/10^6^ cells)	Potentially restricting HLA ^c^
NL**L**TQIGCT	a	WT	L90M	PR	15 (31)	230 (60–484)	B*18
GRNL**L**TQIG	a	WT	L90M	PR	14 (29)	135 (56–290)	C*02
RNL**M**TQIGC	a	DR	L90M	PR	14 (29)	170 (51–450)	A*11, C*04
QHLLRWGL**Y**	a	DR	T215Y	RT	12 (24)	115 (60–200)	C*02, C*15
EELRQHL**L**RW	b	WT	L210W	RT	12 (24)	90 (70–164)	B*39
KMIGG**V**GGFI	b	DR	I50V	PR	11 (22)	90 (51–162)	B*39
ITLWQRP**I**V	b	DR	L10I	PR	10 (20)	169 (56–450)	A*33, C*02, C*16
DTV**I**EEMNL	b	DR	L33I	PR	10 (20)	162 (60–380)	C*02
IGRNL**L**TQI	a	WT	L90M	PR	9 (18)	123 (70–260)	A*26, A*80, B*42, C*17
EK**E**GKISKI	b	WT	E44D	RT	9 (18)	87 (56–190)	A*02, A*80, B*38, B*47
YQY**V**DDLYV	b	DR	M184V	RT	9 (18)	322 (58–1150)	A*01, A*02, B*47
LRWGL**Y**TPD	a	DR	T215Y	RT	8 (16)	99 (58–163)	A*02, A*11
TVL**I**GPTPV	a	DR	V77I	PR	8 (16)	120 (82–200)	A*33, A*74, A*80, B*45, C*16, C*02
DT**IF**EEMNL	b	DR	V32I, L33F	PR	8 (16)	94 (50–190)	A*33, A*74, A*80
LVGPTP**A**NI	b	DR	V82A	PR	8 (16)	87 (50–130)	A*02, A*80, B*39, B*42, B*47, C*17
NTPVFAIK**R**	b	DR	K65R	RT	8 (16)	98 (67–160)	A*80, B*42, C*14 , C*17
**K**KS**V**T**V**LDVGDA**YF**S	b	WT	K103N, V106M, V108I, Y115F, F116Y	RT	8 (16)	223 (51–930)	A*24, A*26
**N**KS**M**T**I**LDVGDA**FY**S	b	DR	K103N, V106M, V108I, Y115F, F116Y	RT	8 (16)	89 (52–119)	A*80, B*15, B*38, B*42, C*17
KQNPDIVI**Y**	b	WT	Y181C	RT	8 (16)	195 (50–523)	A*24, A*26, A*74, A*80, B*42, B*47, C*02, C*17
KQNPDIVI**C**	b	DR	Y181C	RT	8 (16)	170 (60–532)	A*24, A*26, B*39, B*42, C*17
NPDIVI**C**QYM	b	DR	Y181C	RT	8 (16)	116 (60–200)	A*02, A*26, B*27
YQY**M**DDLYV	b	WT	M184V	RT	8 (16)	292 (80–1050)	A*02, A*80, B*18

^a^ A list of the 22 most frequently recognized peptides in the study cohort is shown (>15% of individuals responding). Positions associated with DR are shown in bold.

^b^ According to peptide design rationale as explained in Methods.

^c^ HLA alleles significantly more frequent in individuals responding to the peptide are shown (p<0.01, q<0.2, Mann-Whitney test). HLA molecules that have previously been experimentally linked to the corresponding epitope are shown in bold (Los Alamos HIV Immunology Database). HLA molecules predicted to bind the corresponding epitope using the ELF tool (Los Alamos HIV Immunology Database) are shown in italics.

PR, protease; RT, reverse transcriptase; SFC, spot forming cells.

Of note, for only 3 of the 22 most frequently recognized peptides the previously experimentally confirmed restrictive HLA class I allele was indeed found to be overrepresented in the responder group compared to the individuals that did not mount a response (Table 2). In some cases, HLA alleles predicted to bind the peptide, but not experimentally confirmed to do so were identified as well. Interestingly, in some of these latter cases, the response was associated to HLA class I alleles frequently observed in populations with Amerindian ancestry (such as A*24, B*39, C*04), or with alleles with higher frequency in Caucasian populations compared to Amerindian populations (A*11, B*42, C*17, C*02) [23]. Interestingly, responses to peptides overlapping statistically predicted HLA-associated DR sites [13] (7/22 most frequently recognized peptides, Table 2) generally did not match the anticipated HLA defining the association. These observations suggest a level of HLA promiscuity in the recognition of the peptides.

### Response patterns to DR peptides are not associated with the presence of DR mutations in the circulating viral population

Due to the frequent recognition of peptides containing DR mutations in the study cohort, the presence of DR variants that could have stimulated these responses was investigated. As all subjects in the study were ART-naïve, the frequency of DR mutations was expected to be low [24]. We conducted a detailed deep sequencing analysis of the circulating virus population in individuals from CIENI for whom plasma samples were available. Sequences were obtained for 20 of 24 CIENI subjects with a median sequencing depth of 645 (IQR 315–982, after filtering reads by size and quality). Only reads with size >100 bases and mean quality >Q30 were considered. We then assessed whether the presence and frequency of individual DR mutations after alignment to the HXB2 consensus explained the detection of DR-sequence specific responses. DR variants and polymorphisms in DR positions were observed in 12 of the 20 participants assayed (S2 Table). Nevertheless, in no case did the presence of the DR variant correspond to the presence of ELISpot response to the DR variants. Interestingly, 4 cases of response to DR peptides overlapping the RT K103 position were observed in which the DR variant K103R, but not K103N was observed in the viral population, suggesting a possible cross-reactive effect (S2 Table). Taken together, these observations suggest that the presence of DR variants, even at low levels, does not explain the presence of ELISpot responses to peptides containing different DR mutations.

To further assess whether the presence of specific DR or WT sequences predicted the detection of specific responses we compared ELISpot response patterns and the existence of the corresponding epitope in the viral quasiespecies in each participant (Table 3). Interestingly, the mean proportion of responses to WT peptides that coincided with the presence of the corresponding epitope was 68% of cases, while the mean proportion of responses to DR peptides that coincided with the presence of the corresponding epitope was only 1.4% (p = 0.0002). Considering each individual case, the proportion of responses to WT peptides in the presence of the corresponding WT epitope in the patient virus was significantly higher than the proportion of responses to DR peptides in the presence of the corresponding DR epitope in 9 of 17 (53%) participants. In most of the remainder cases the number of responses was not sufficient to observe significant differences. Similar results were observed when separating the analysis for peptides belonging to Set “a” (S3 Table) and Set “b” (S4 Table). These observations suggest that, although a large proportion of responses to WT peptides can be explained by the presence of the epitope in the patient plasma virus, responses to DR variants in general do not coincide with the presence of the corresponding sequence in the circulating virus.

**Table 3:** Correlation between immunological response to specific peptides and presence of the corresponding epitope in the patient virus ^a^.

Patient	Total number of peptides with response	Total number of WT peptides with response	Total number of DR peptides with response	Responses to WT peptide with presence of WT epitope [n (%)]	Responses to WT peptide w/o presence of WT epitope [n (%)]	Responses to DR peptide with presence of DR epitope [n (%)]	Responses to DR peptide w/o presence of DR epitope [n (%)]	p
M24	47	13	34	8 (62)	5 (38)	0	34 (100)	<0.0001
M25	41	17	24	11 (65)	6 (35)	1 (4)	23 (96)	<0.0001
M26	7	2	5	1 (50)	1 (50)	0	5 (100)	NS
M28	18	8	10	5 (63)	3 (38)	0	10 (100)	0.0065
M30	64	24	40	17 (71)	7 (29)	2 (5)	38 (95)	<0.0001
M31	35	14	21	9 (64)	5 (36)	1 (5)	20 (95)	0.0002
M32	34	19	15	4 (21)	15 (79)	0	15 (100)	NS
M35	9	1	8	1 (100)	0	0	8 (100)	NS
M36	5	1	4	1 (100)	0	0	4 (100)	NS
M37	44	25	19	19 (76)	6 (24)	0	19 (100)	<0.0001
M38	16	5	11	2 (40)	3 (60)	0	11 (100)	NS
M39	64	29	35	28 (97)	1 (3)	0	35 (100)	<0.0001
M40	24	5	19	5 (100)	0	0	19 (100)	<0.0001
M45	1	0	1	0	0	0	1 (100)	NS
M46	2	0	2	0	0	0	2 (100)	NS
M47	40	11	29	7 (64)	4 (36)	0	29 (100)	<0.0001
M48	2	1	1	1 (100)	0	0	1 (100)	NS
Mean	26.6	10.3	16.4	7 (68)	3.3 (32)	0.2 (1.4)	16.1 (96.6)	0.0002

^a^ The presence or absence of the complete WT or DR sequence corresponding to the assayed peptides was assessed by moving window analyses from NGS data for each patient as described in Methods. A threshold of 2% was used to determine the presence or absence of the WT or DR epitope (see Methods).

## Discussion

We have explored the possibility of limiting HIV evolution *in vivo* by the concerted selective pressures exerted by the cellular immune response and ART on the virus’ Pol region. By assessing responses to WT-DR peptide pairs overlapping positions where DR mutations occur on predicted or defined HLA class I-restricted epitopes, we observed that responses to peptides containing DR mutations were frequent and strong in a cohort of ART-naïve individuals. Indeed, peptides including DR mutations were among the most frequently recognized in the study cohort and their recognition was not explained by the presence of DR variants in the viral population, even when considering low frequency sequence variants. This analysis allowed the identification of candidate peptides that contain DR mutations and which could provide a benefit for patients under ART when used in a putative therapeutic vaccine, given that they could both reinforce viral suppression and prevent the development of drug resistance. These peptides could also be useful as immunogens in kick and kill strategies, where the virus is being reactivated after therapeutic vaccination, but still while the individual is under ART, although further experiments are warranted to confirm peptide immunogenicity in individuals under ART. It is important to note that few positions in PR and RT, which are associated with DR have also shown association with HLA [22]. An interesting point raised in the present study is that, although a direct HLA association with the DR position has not been identified in most cases, the presence of the DR variant is sufficient to promote ELISpot responses, and that these responses are frequently exclusive to the DR peptide or to the WT peptide only. Additionally, it could be argued that looking at DR positions in negative association with HLA would be relevant, as they would suggest specific recognition of the DR variant and conservation of the WT amino acid. However, among the 8 HLA-associations previously described for DR mutation sites in ART-naïve Caucasian individuals [22], only one was a negative association (PR A71 with B*57:01). Additionally, in an older study on a cohort of Australian individuals failing ART [13], 6 DR mutation sites in RT were reported to be associated with HLA, all positive associations. Only RT L210W overlaps between the two studies.

The study cohort was composed half by individuals of Mexican origin and half by individuals of Caucasian origin. Demographic and clinical characteristics of both cohorts were similar. Nevertheless, HLA frequency distributions differed between them (S1 Dataset), as expected when comparing individuals of Caucasian origin to individuals with Amerindian ancestry component [23]. Even considering these differences, magnitude of response, as well as response frequency did not vary between the two cohorts. Neither were specific peptides exclusively recognized by individuals in either one of the cohorts, allowing us to analyse the two cohorts as one. Although the median number of peptides recognized per individual was 16, there were some patients for whom an unexpectedly high number of responses were observed: 71, 122 and 123 peptides recognized with HLA genotypes B*57/B*18, B*1503/B*44:03 and B*15:16/B*42:01 respectively. These responses were confirmed and backgrounds were comparable to those observed in other responders. These unusual responses coincided with the recognition of most peptide sets overlapping single regions of the gene. It is important to note that peptides were designed independently of the HLA repertoire of the participants of the study cohort. Considering the presence of the restrictive HLA of the optimally defined epitopes included in the participants, we would expect at least 47% (23) of patients responding to at least one peptide. Surprisingly, the frequency of responses was much higher than that, with 82% (40) patients responding to at least one peptide (S1 Dataset). It is also interesting to consider, that several responses were not associated with the expected restrictive HLA allele, possibly indicating recognition promiscuity [25, 26] or unknown HLA specificities of poorly described HLA alleles characteristic of Amerindian populations.

Unexpectedly, we observed 22 peptides that were recognized by more than 15% of the participants, from which 14 (64%) included DR mutations (Table 3). This proportion was conserved (67% of 134 were DR peptides) when going down to a recognition threshold of 10% of the individuals in the cohort. The high recognition frequency of these peptides in an immunogenetically diverse cohort makes them good immunogen candidates for putative therapeutic vaccines and supports the idea that cornering the virus between concerted immunologic and pharmacologic selective pressures might be possible. In particular, cases in which a therapeutic vaccine including DR peptides could reinforce ART in a virally suppressed patient, additionally preventing the development of drug resistance, come to mind. In particular, peptides containing highly relevant mutations for HIVDR including K65R, M184V, T215Y for NRTI; K103N, Y181C for NNRTI; and V32I, I50V, V82A, L90M, for PI were frequently recognized (Table 3).

On the other hand, we also observed several WT peptide-specific responses with high frequency of recognition and with strong median magnitudes of response. When comparing the response of WT and DR peptide pairs, we observed a median of 4 fully cross reactive responses per individual for WT peptides only (Fig 3). This suggests that the presence of the DR mutation in the peptide could also serve as a CTL escape variants, effectively abrogating CTL recognition. Indeed, 3 of the 8 most frequently recognized peptides in the cohort were WT (Fig 4). In these cases, knowing the HLA of the patient would be useful to avoid, as far as possible, the use of antiretroviral drugs that could promote the double viral escape, e. g. The peptide NL**L**TQIGCT overlapping the protease L90 position was the most frequently recognized in the study cohort (Table 3), with a significantly higher median magnitude of response compared to the DR version (Table 1). This epitope is restricted by HLA-B*18. Thus, patients with this HLA, treated with protease inhibitors would be more prone to develop the L90M DR (and in this case, also immune escape) mutation.

It has been previously proposed, that a CD8+ T cell response-associated “original antigenic sin” effect could exist with the elicitation of pre-existing lower avidity cross-reactive memory T cells in the case of a secondary exposure to some highly variable pathogens, including dengue viruses and human papilloma viruses [27–29]. The expansion of cross-reactive memory T cells with low avidity for epitopes of the variant responsible for the second antigenic exposure over naïve T cells with higher avidity for the new variant would result in low magnitude and/or suboptimal T cell responses. Interestingly, in the present study, the median magnitude of response observed for DR peptides did not differ to that observed for WT peptides (Table 2). Also of note is the fact that several of the peptides with the highest frequency of recognition, also showed significantly higher magnitudes of response to DR peptides (Tables 1 and 3), e.g. the PR epitope RNL**M**TQIGC, including L90M, and the RT epitope QHLLRWGL**Y**, including T215Y, both showed recognition frequency of 29% and a significantly higher median Δ DR-WT magnitude of response. This observation suggests that in general, responses observed to DR peptides were not the result of weak cross-reactive effects, but rather independently generated responses. Indeed, a restricted T cell receptor (TCR) promiscuity has been described for variant epitopes with a limited degree of tolerance for epitope variation in the elicitation of strong CTL responses [30]. In the work by Hoof et al., peptides eliciting ELISpot responses were found to be significantly more similar to the autologous virus than those that did not elicit a response, with a single substitution in the peptide decreasing the chance of observing a CTL response by 40%. A similar effect was observed in our study, in which responses elicited by WT peptides coincided with the presence of the epitope in the autologous virus in a median 73% of cases. Interestingly however, responses to DR peptides did not coincide with the presence of the DR epitope in the autologous plasma virus, even when looking at low frequency variants. Several scenarios could explain this effect. First, the introduction of DR mutations in some peptides had no visible effect on peptide recognition by CTL, showing a degree of TCR promiscuity to peptides in which the introduction of the DR mutation might not abrogate binding to the restricting HLA molecule, as previously described [30]. Second, novel epitopes containing DR mutations could exist, with no immunogenicity observed to the WT peptide, especially considering that the HLA repertoire in the study cohort included several incompletely described HLA alleles mainly of Amerindian origin. Third, even when our method did not allow us to find DR variants in the autologous virus, which could explain the presence of responses to DR peptides, DR variants could still exist in lymphoid tissue or other body compartments, they could be present in lower frequencies that our method was able to detect with confidence (<2%), or they could have spontaneously appeared in previous time points causing cross reactivity. Fourth, indeed, an original antigenic sin effect could exist for some DR peptides, eliciting cross-reactive responses that, although of similar magnitudes compared to responses to WT peptides, could qualitatively be different from WT responses [31]. Further studies are needed to assess the quality of CTL responses elicited by these highly recognized DR peptides, including T cell polyfunctionality and patterns of cytokine production, in order to support their possible role as effective immunogens in combined immunologic-pharmacologic HIV control strategies. Detailed TCR analyses of responses directing responses to WT and/or DR sequences may also help to understand the ontogeny of these responses. Furthermore, to support the possible use of DR peptides in therapeutic approaches, their immunogenicity in putative target patient groups including individuals under ART and individuals with ART failure who have developed DR will have to be assessed. Additionally, the study of peptides from other therapeutic targets such as the integrase and Gag proteins may increase therapeutic options.

## Conclusion

Our observations suggest that cornering HIV through the immune recognition of DR variants could be feasible, especially when based on the use of frequently recognized and strongly reactive peptides. Nevertheless, we also found several cases of HIV double escape, where the presence of a DR mutation rendered a peptide non immunogenic, while the WT version of the peptide showed response. The use of opposing selective pressures on HIV could be useful to design personalized combined treatment and vaccination strategies, avoiding ART regimens prone to failure by the influence of the immune response and promoting the use of ARV drugs associated with the selection of DR variants recognized by the immune response in the immunogenetic context of the treated patient. Our work suggests that the use of DR peptides in putative therapeutic vaccines, could not only support viral suppression, but also avoid the development of DR, providing a double advantage for the patient.

Although our study has caveats that limit our conclusions, our results suggest as proof of principle that DR peptides are unexpectedly immunogenic and attention should be given to their possible use in HIV control strategies. Further studies are needed to assess the quality of CTL responses elicited by DR peptides, as well as their frequency of recognition in cohorts with different immunogenetic contexts. Also, the inclusion of putative target patient groups such as individuals under suppressive ART or with ART failure will be necessary to assess the viability of the inclusion of DR peptides in therapeutic strategies. Additionally, it will be interesting to extend these studies to other HIV proteins that could function as possible therapeutic targets, including the integrase and Gag.

## Supporting Information

S1 Dataset ELISpot response magnitudes to independent peptides per study participant and HLA allele distributions. (XLSX)

S1 Fig Wide spectrum of differential magnitudes of response to WT and DR peptide pairs divided by peptide Set. Differential magnitudes of response to each DR-WT peptide pair (Δ) are shown in the upper panels for each responder separating peptide Sets “a” and “b” (for a description of each peptide design see Methods). Δ were calculated as the magnitude of response (SFC/mllion PBMC) to the DR sequence minus the magnitude of response to the WT sequence in each subject individually. A median differential magnitude was then calculated across all individuals responding to either the WT, the DR or both sequences. Boxes represent 50% of differential magnitude to each DR-WT peptide pair, whiskers maximum and minimum of the differential magnitude. In the lower panels, only median Δ responses are shown. Peptide pairs with significantly higher differential response to DR are shown in red; peptide pairs with significantly higher differential response to WT are shown in blue (p<0.05).
(TIFF)

S2 Fig Recognition frequency of DR mutations by peptide set. ELISpot responses to a panel of 45 and 95 DR-WT peptide pairs for Set “a” and Set “b” respectively, were assessed in 49 individuals (for a description of each of the two peptide design approaches see Methods). The number of peptide pairs for which, both the WT and DR, only DR, or only WT peptides were recognized was assessed per individual. The scatter plot shows the result of three Wilcoxon tests comparing the paired data between groups, significant p values are indicated. Error bars show the median with interquartile range.
(TIFF)

S3 Fig Recognition frequency and differential magnitudes of response to DR and WT peptides overlapping DR mutations with and without HLA association. ELISpot responses to a panel of 39 and 56 DR-WT peptide pairs for peptides overlapping DR positions with and without HLA association respectively were assessed in 49 individuals. The presence of absence of HLA associations was determined by comparison to previously published data [22]. The number of peptide pairs for which, both the WT and DR, only DR, or only WT peptides were recognized was assessed per individual. The scatter plot shows the result of three Wilcoxon tests comparing the paired data between groups, significant p values are indicated. Error bars show the median with interquartile range. Δ were calculated as the magnitude of response (Spot-Forming Cells/mllion PBMC) to the DR sequence minus the magnitude of response to the WT sequence in each subject individually for each peptide pair. Boxes represent 50% of differential magnitude to each DR-WT peptide pair, whiskers maximum and minimum of the differential magnitude. Peptide pairs with significantly higher differential response to DR are shown in red; peptide pairs with significantly higher differential response to WT are shown in blue (p<0.05).
(TIFF)

S1 Table Median magnitudes of ELISpot responses to WT and DR peptides for both peptide sets. (DOCX)

S2 Table Presence of DR mutations overlapped by the assayed peptides per individual in the study cohort. (DOCX)

S3 Table Correlation between immunological response to specific peptides and presence of the corresponding epitope in the patient virus for peptide Set “a”. (DOCX)

S4 Table Correlation between immunological response to specific peptides and presence of the corresponding patient virus for peptide Set “b”. (DOCX)
