# Correction: Identification of Immunogenic Cytotoxic T Lymphocyte Epitopes Containing Drug Resistance Mutations in Antiretroviral Treatment-Naïve HIV-Infected Individuals

**DOI:** 10.1371/journal.pone.0153340

**Published:** 2016-04-05

**Authors:** Juan Blanco-Heredia, Aarón Lecanda, Humberto Valenzuela-Ponce, Christian Brander, Santiago Ávila-Ríos, Gustavo Reyes-Terán

The following information is missing from the Acknowledgments section: JBH is a doctoral student from Programa de Doctorado en Ciencias Biomédicas, Facultad de Medicina, Universidad Nacional Autónoma de México (UNAM), and received fellowship 229424 from CONACyT.
