# Supplementary figures and images for: Identification of Immunogenic Cytotoxic T Lymphocyte Epitopes Containing Drug Resistance Mutations in Antiretroviral Treatment-Naïve HIV-Infected Individuals
Source: PLoS One. 2016 Jan 25;11(1):e0147571. doi: 10.1371/journal.pone.0147571 (PMC4725752; doi:10.1371/journal.pone.0147571)

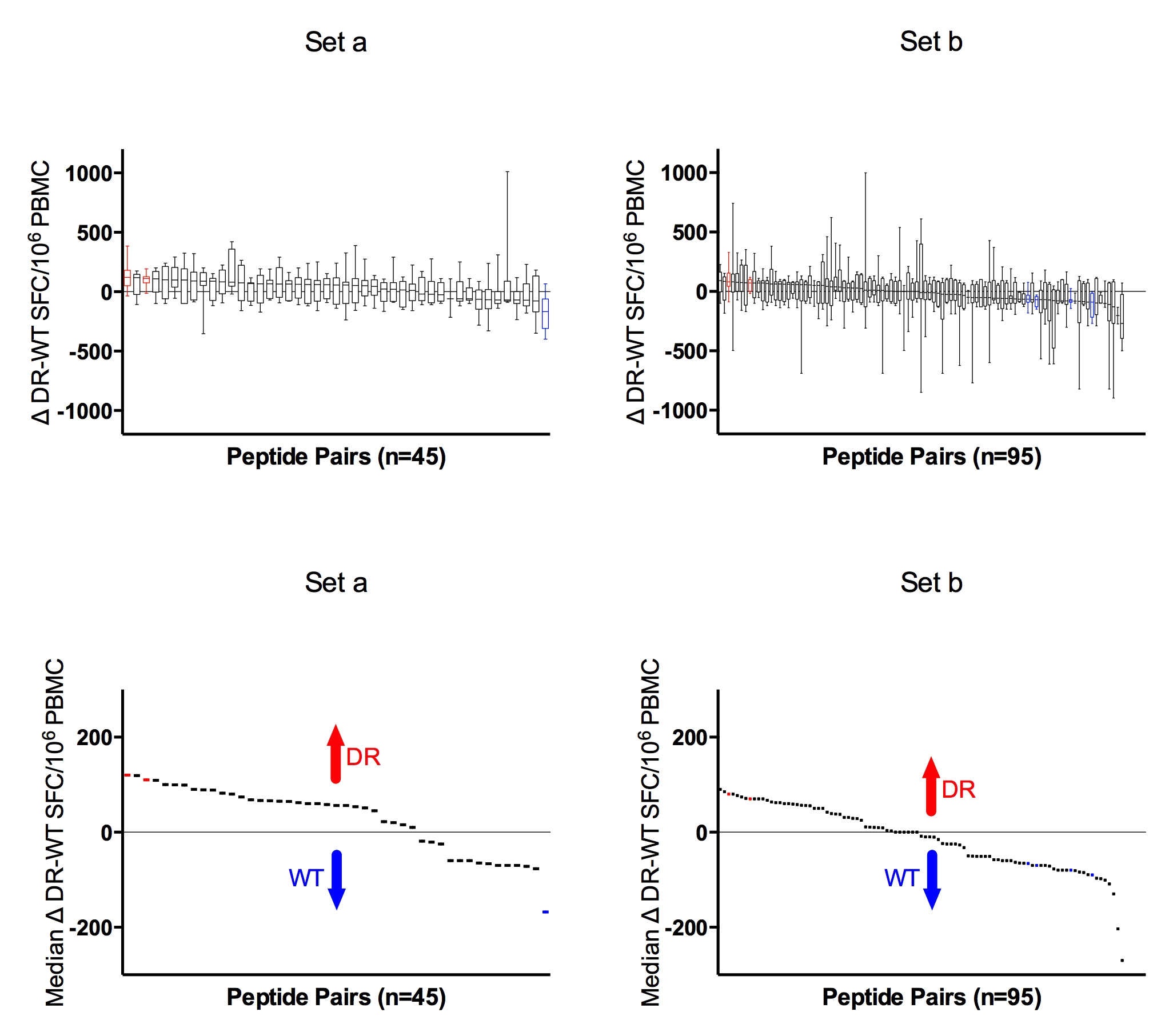

Supplement: S1 Fig — Differential magnitudes of response to each DR-WT peptide pair (Δ) are shown in the upper panels for each responder separating peptide Sets “a” and “b” (for a description of each peptide design see Methods). Δ were calculated as the magnitude of response (SFC/mllion PBMC) to the DR sequence minus the magnitude of response to the WT sequence in each subject individually. A median differential magnitude was then calculated across all individuals responding to either the WT, the DR or both sequences. Boxes represent 50% of differential magnitude to each DR-WT peptide pair, whiskers maximum and minimum of the differential magnitude. In the lower panels, only median Δ responses are shown. Peptide pairs with significantly higher differential response to DR are shown in red; peptide pairs with significantly higher differential response to WT are shown in blue (p<0.05). (TIFF) [file pone.0147571.s002.tiff]

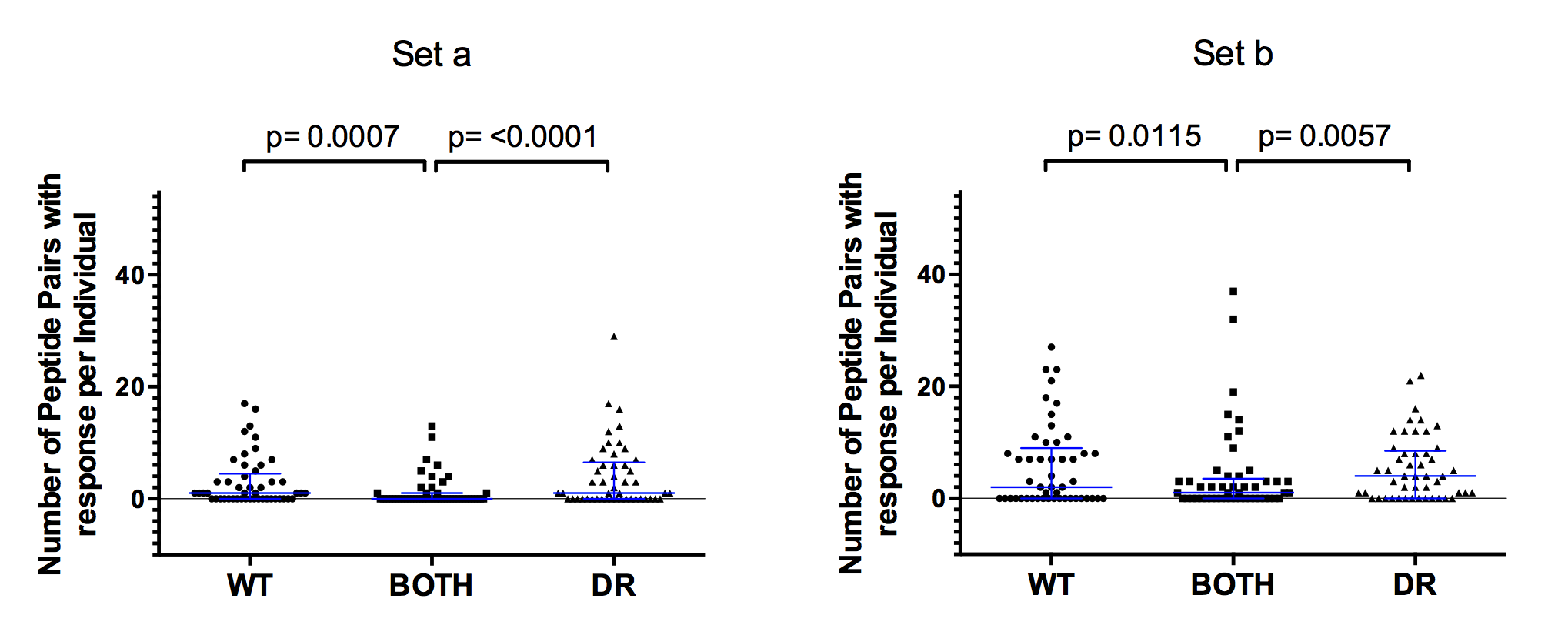

Supplement: S2 Fig — ELISpot responses to a panel of 45 and 95 DR-WT peptide pairs for Set “a” and Set “b” respectively, were assessed in 49 individuals (for a description of each of the two peptide design approaches see Methods). The number of peptide pairs for which, both the WT and DR, only DR, or only WT peptides were recognized was assessed per individual. The scatter plot shows the result of three Wilcoxon tests comparing the paired data between groups, significant p values are indicated. Error bars show the median with interquartile range. (TIFF) [file pone.0147571.s003.tiff]

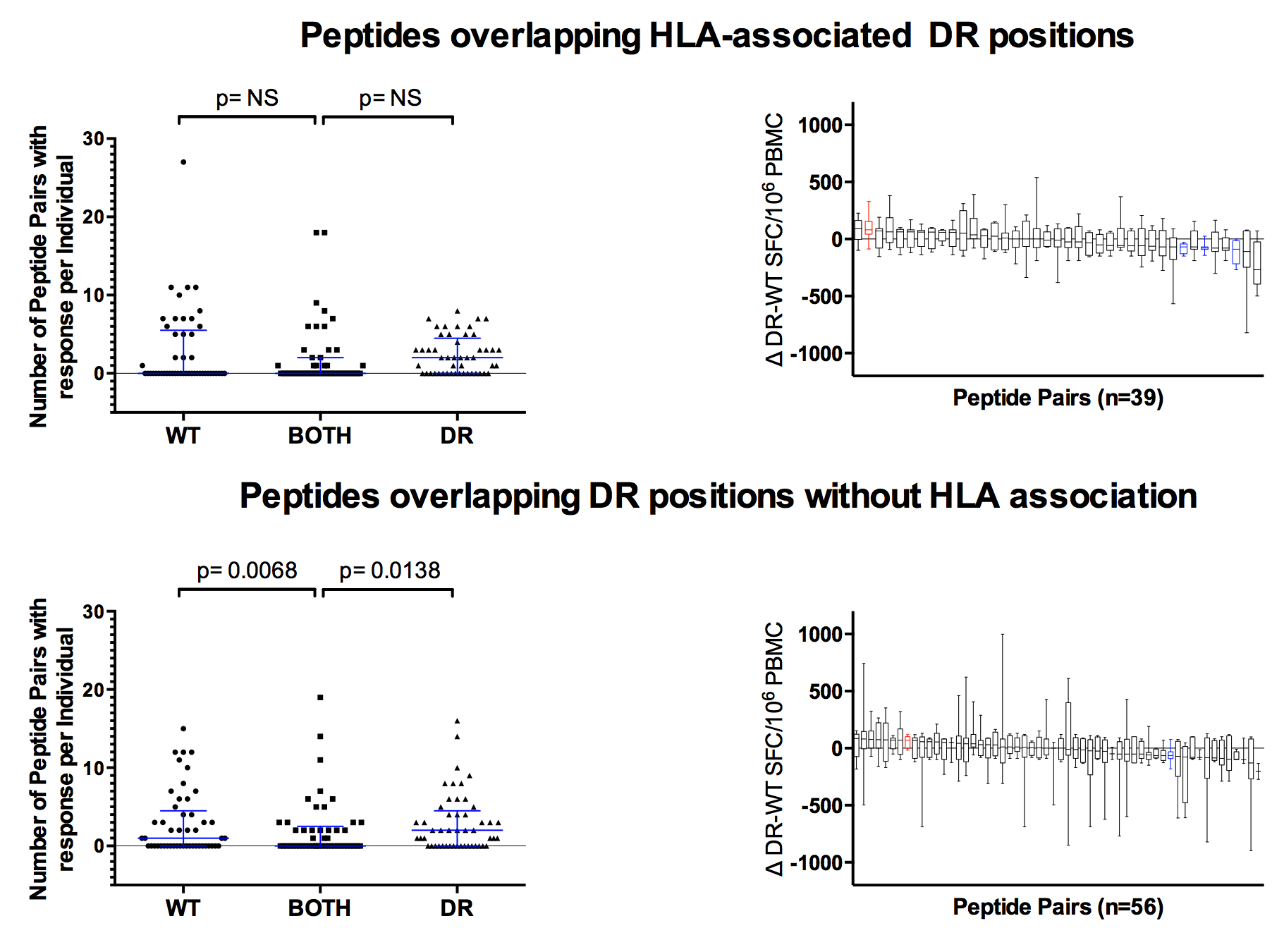

Supplement: S3 Fig — ELISpot responses to a panel of 39 and 56 DR-WT peptide pairs for peptides overlapping DR positions with and without HLA association respectively were assessed in 49 individuals. The presence of absence of HLA associations was determined by comparison to previously published data [22]. The number of peptide pairs for which, both the WT and DR, only DR, or only WT peptides were recognized was assessed per individual. The scatter plot shows the result of three Wilcoxon tests comparing the paired data between groups, significant p values are indicated. Error bars show the median with interquartile range. Δ were calculated as the magnitude of response (Spot-Forming Cells/mllion PBMC) to the DR sequence minus the magnitude of response to the WT sequence in each subject individually for each peptide pair. Boxes represent 50% of differential magnitude to each DR-WT peptide pair, whiskers maximum and minimum of the differential magnitude. Peptide pairs with significantly higher differential response to DR are shown in red; peptide pairs with significantly higher differential response to WT are shown in blue (p<0.05). (TIFF) [file pone.0147571.s004.tiff]
