# Supplementary material for: Identification of Immunogenic Cytotoxic T Lymphocyte Epitopes Containing Drug Resistance Mutations in Antiretroviral Treatment-Naïve HIV-Infected Individuals
Source: PLoS One. 2016 Jan 25;11(1):e0147571. doi: 10.1371/journal.pone.0147571 (PMC4725752; doi:10.1371/journal.pone.0147571)
Supplement: S1 Table — (DOCX) [file pone.0147571.s005.docx]

**S1 Table. Median magnitudes of ELISpot responses to WT and DR peptides for both peptide sets**

|  | **Both peptide sets** | | | **Peptide Set a** | | | **Peptide Set b** | | |
| --- | --- | --- | --- | --- | --- | --- | --- | --- | --- |
|  | Median | (IQR) | | Median | (IQR) | | Median | (IQR) | |
| Responses to all peptides (SFC/10^6^ PBMC) | 95.6 | (81, | 113) | 99.5 | (72.9, | 150) | 90 | (71, | 130) |
| Only responses to WT peptides (SFC/10^6^ PBMC) | 94.8 | (80, | 104.7) | 91.2 | (72.9, | 163.3) | 91.2 | (72.9, | 150) |
| Only responses to DR peptides (SFC/10^6^ PBMC) | 99.4 | (81.1, | 115.9) | 100.3 | (72.9, | 127.7) | 90 | (70, | 129) |
| p ^a^ | 0.2844 | | | 0.0125* | | | 0.6995 | | |

^a^ Mann-Whitney test between responses to WT peptides only vs. DR peptides only
