# Supplementary material for: Identification of Immunogenic Cytotoxic T Lymphocyte Epitopes Containing Drug Resistance Mutations in Antiretroviral Treatment-Naïve HIV-Infected Individuals
Source: PLoS One. 2016 Jan 25;11(1):e0147571. doi: 10.1371/journal.pone.0147571 (PMC4725752; doi:10.1371/journal.pone.0147571)
Supplement: S2 Table — (DOCX) [file pone.0147571.s006.docx]

**S2 Table. Presence of DR mutations overlapped by the assayed peptides per individual in the study cohort ^a^**

|  | **M28** | **M30** | **M37** | **M39** | **M40** | **M41** | **M43** | **M44** | **M45** | **M46** | **M47** | **M48** |
| --- | --- | --- | --- | --- | --- | --- | --- | --- | --- | --- | --- | --- |
| PR L10I |  |  |  |  |  |  | 10.3 |  |  |  |  |  |
| PR K20R ^b^ |  |  |  |  | 1.1 |  |  |  |  |  |  |  |
| PR D30N |  |  |  |  |  |  | 1.2 |  |  |  |  |  |
| PR L33V ^b^ |  |  |  |  |  |  |  |  |  |  | **3.6** |  |
| PR V82I ^b^ |  |  |  |  |  |  |  | 99.5 |  |  |  | 2.5 |
| RT D67N |  |  |  |  |  | 93.2 |  |  |  |  |  |  |
| RT K103R ^b^ |  | **97.7** | 98.3 | **4.6** | **7.1** |  | 5.7 | 2.9 | 7.3 | 3.9 | **7.5** | 3.9 |
| RT V108I | 4.6 |  |  |  |  |  |  |  |  |  |  |  |
| RT V118I |  |  |  |  |  |  |  |  | 25.6 |  |  |  |
| RT K219Q |  |  |  |  |  | 99.5 |  |  |  |  |  |  |

^a^ The proportion of HIVDR variants in the viral population was assessed by NGS for each participant as described in Methods. Percentages of viruses within the viral population carrying each DR mutation in each participant are shown. Only DR mutations overlapped by the assayed peptides and found in at least one participant are shown. Only individuals in which DR variants were found are shown. Cases of coincidence between ELISpot response to DR peptides and presence of the corresponding DR variant are marked in bold.

^b^ Variants included in the peptides tested for these DR positions did not correspond to the DR variant found in the virus.
