# Supplementary material for: Identification of Immunogenic Cytotoxic T Lymphocyte Epitopes Containing Drug Resistance Mutations in Antiretroviral Treatment-Naïve HIV-Infected Individuals
Source: PLoS One. 2016 Jan 25;11(1):e0147571. doi: 10.1371/journal.pone.0147571 (PMC4725752; doi:10.1371/journal.pone.0147571)
Supplement: S4 Table — (DOCX) [file pone.0147571.s008.docx]

**S4 Table. Correlation between immunological response to specific peptides and presence of the corresponding epitope in the patient virus for peptide Set “b” ^a^**

| Patient | Total number of peptides with response | Total number of WT peptides with response | Total number of DR peptides with response | Responses to WT peptide with presence of WT epitope | Responses to WT peptide w/o presence of WT epitope | Responses to DR peptide with presence of DR epitope | Responses to DR peptide w/o presence of DR epitope | p |
| --- | --- | --- | --- | --- | --- | --- | --- | --- |
|  |  |  |  | [n (%)] | [n (%)] | [n (%)] | [n (%)] |  |
| M24 | 33 | 7 | 26 | 4(57) | 3(43) | 0 | 26(100) | 0.0009 |
| M25 | 18 | 4 | 14 | 2(50) | 2(50) | 0 | 14(100) | 0.0392 |
| M26 | 6 | 1 | 5 | 0 | 1(100) | 0 | 5(100) | NS |
| M28 | 16 | 6 | 10 | 3(50) | 3(50) | 0 | 10(100) | 0.0357 |
| M30 | 48 | 16 | 32 | 9(56) | 7(44) | 0 | 32(100) | <0.0001 |
| M31 | 20 | 7 | 13 | 3(43) | 4(57) | 1(8) | 12(92) | NS |
| M32 | 17 | 9 | 8 | 2(22) | 7(78) | 0 | 8(100) | NS |
| M35 | 8 | 1 | 7 | 1(100) | 0 | 0 | 7(100) | NS |
| M36 | 5 | 1 | 4 | 1(100) | 0 | 0 | 4(100) | NS |
| M37 | 10 | 4 | 6 | 3(75) | 1(25) | 0 | 6(100) | 0.0333 |
| M38 | 10 | 2 | 8 | 1(50) | 1(50) | 0 | 8(100) | NS |
| M39 | 39 | 12 | 27 | 11(92) | 1(8) | 0 | 27(100) | <0.0001 |
| M40 | 16 | 2 | 14 | 2(100) | 0 | 0 | 14(100) | 0.0083 |
| M45 | 0 | 0 | 0 | 0 | 0 | 0 | 0 | NS |
| M46 | 2 | 0 | 2 | 0 | 0 | 0 | 2(100) | NS |
| M47 | 39 | 10 | 29 | 6(60) | 4(40) | 0 | 29(100) | <0.0001 |
| M48 | 1 | 0 | 1 | 0 | 0 | 0 | 1(100) | NS |
| **Mean** | **16.9** | **5.1** | **12.9** | **3(50.3)** | **2.1(32.1)** | **0.1(0.5)** | **12.1(93.7)** | **0.0147** |

^a^ The presence or absence of the complete WT or DR sequence corresponding to the assayed peptides was assessed by moving window analyses from NGS data for each patient as described in Methods. A threshold of 2% was used to determine the presence or absence of the WT or DR epitope.
